# Clinical Utility of a Targeted Next-Generation Sequencing Panel for Inherited Platelet Disorders in Children

**DOI:** 10.3390/diagnostics15172210

**Published:** 2025-08-30

**Authors:** Dilek Kaçar, Mustafa Altan, Turan Bayhan, Said Furkan Yıldırım, Fatma Burçin Kurtipek, Özlem Arman Bilir, Namık Yaşar Özbek, Neşe Yaralı

**Affiliations:** 1 Department of Pediatric Hematology Oncology, Ankara Bilkent City Hospital, University of Health Sciences, Ankara 06800, Turkey; ozlemarman@gmail.com (Ö.A.B.); namikyozbek@gmail.com (N.Y.Ö.); 2 Department of Medical Genetics, Ankara Bilkent City Hospital, Ankara 06800, Turkey; mustafaltan@gmail.com (M.A.); saidfurkanyildirim@gmail.com (S.F.Y.); 3 Department of Pediatric Hematology Oncology, Ankara Bilkent City Hospital, Ankara Yıldırım Beyazıt University, Ankara 06800, Turkey; turanbayhan@yahoo.com (T.B.); burcindogan86@gmail.com (F.B.K.); neseyarali@yahoo.com (N.Y.)

**Keywords:** genetic diagnosis, inherited platelet disorders, next-generation sequencing, pediatric patients, thrombocytopenia

## Abstract

**Background/Objectives**: Inherited platelet disorders (IPDs) are diverse conditions characterized by abnormalities in platelet count and function. Next-Generation Sequencing (NGS) shows promise as a diagnostic tool in the diagnosis of IPDs. This study aims to assess the clinical value and limitations of using a targeted NGS panel in diagnosing children with suspected IPDs. **Methods**: We conducted a retrospective study of 93 children evaluated for suspected IPDs. A targeted NGS panel of 14 IPD-associated genes (*RUNX1*, *WAS*, *ADAMTS13*, *ANKRD26*, *CYCS*, *GATA1*, *GP1BA*, *GB1BB*, *GP9*, *ITGA2B*, *ITGB3*, *MASTL*, *MPL*, *MYH9*) was performed. **Results**: Genetic variants were identified in 30 patients (32.3% of the cohort). A total of 37 variants, of which 15 (40.5%) were novel, were found across 11 of the 14 genes on the panel (all except *MPL*, *CYCS*, and *RUNX1*). Variants were most frequently found in *ITGB3* (18.9% of variants), *GP1BA* (16.2%), and *ADAMTS13* (16.2%) genes. The majority of variants (64.9%) were classified as variants of uncertain significance (VUS), followed by likely pathogenic (LP) (27%) and pathogenic (8.1%) variants. Most variants were in a heterozygous state (73%). Specific cases highlighted complex genetic scenarios, such as co-occurring variants, and the identification of pathogenic and LP variants in patients initially presenting with immune thrombocytopenia. **Conclusions**: NGS helps to identify genetic causes, assess risk, manage, and provide genetic counseling in the management of IPDs. However, the prevalence of VUS underscores the need for a multidisciplinary approach to evaluate NGS results accurately.

## 1. Introduction

Inherited platelet disorders (IPDs) are a highly varied group characterized by abnormalities in platelet count and/or function. While the clinical presentation ranges from minor mucocutaneous bleeding to severe, life-threatening hemorrhage, many IPDs are also part of multisystemic disorders called syndromic IPDs. In these cases, patients may face an increased risk of developing other organ problems or cancers, making accurate diagnosis essential for proper risk assessment and management [1]. The exact prevalence of IPDs is not well known; a database study found that 0.329% of the general population carries a clinically significant predicted loss-of-function variant in a platelet-related gene [2]. Additionally, in areas with high rates of consanguinity, the prevalence of rare autosomal recessive (AR) IPDs is notably higher [3].

Historically, diagnosing IPDs has been a significant challenge due to their extensive clinical and laboratory heterogeneity [4]. The diagnostic workup of IPDs includes a detailed history of bleeding, a family history of thrombocytopenia or other malignancies, and a thorough physical examination to identify the potential syndromic IPD features. Routine laboratory evaluations, including complete blood count, peripheral blood smear (PBS), prothrombin time (PT), activated partial thromboplastin time (aPTT), and screening tests for von Willebrand disease, are crucial [1,4]. Laboratory tests such as light transmission aggregometry (LTA) for platelet functions and flow cytometric analysis of major platelet adhesive glycoproteins often lack sensitivity, are technically demanding, or have limited applicability, particularly in pediatric patients. As a result, many IPD patients remain undiagnosed or misdiagnosed into adulthood, which increases the risk of inappropriate clinical interventions.

The increasing accessibility and decreasing cost of Next-Generation Sequencing (NGS) technologies have transformed the traditional diagnostic paradigm, enabling the simultaneous sequencing of multiple genes associated with a specific phenotype. This has led to a growing advocacy for the earlier integration of molecular studies into diagnostic algorithms [4]. Despite these advancements, the clinical utility of genetic testing for IPDs and the precise algorithm for its integration into the diagnostic workflow remain subjects of ongoing controversy.

In this study, we aimed to address this gap by comprehensively evaluating the clinical value and limitations of integrating a targeted NGS panel into the diagnostic workflow for a cohort of pediatric patients with suspected IPDs.

## 2. Materials and Methods

This retrospective observational study involved pediatric patients under 18 years old evaluated between 1 August 2019 and 31 March 2025. Patients were identified as having suspected IPDs based on a combination of clinical signs, characteristic laboratory findings, and a positive family history of similar bleeding symptoms and/or thrombocytopenia. The study included individuals with chronic thrombocytopenia, often accompanied by elevated mean platelet volume (MPV), regardless of bleeding history. Patients with a history of bleeding but normal platelet counts were also included. Key laboratory indicators were normal coagulation studies, specific abnormalities in platelet morphology (such as giant or small platelets noted on a PBS), reduced surface glycoprotein expression determined by flow cytometry, and, in select cases, decreased ADAMTS13 enzymatic activity. ADAMTS-13 activity was measured at a contracted private laboratory using a validated assay. According to the laboratory’s protocol, if activity was below 40%, ADAMTS-13 inhibitor and antigen tests were performed reflexively. Patients with transient thrombocytopenia, thrombocytopenia due to secondary causes such as hypersplenism or bone marrow failure syndromes, other inherited coagulation disorders, or acquired bleeding causes, such as anticoagulant use, severe liver or kidney disease, vitamin K deficiency, or disseminated intravascular coagulation, were excluded. A targeted NGS panel specific for IPDs was performed on these patients during the study.

Collected data included demographic characteristics, severity of bleeding, indications for NGS testing, platelet counts, MPV values, presence of pre-existing cytopenia, PBS findings, PT and aPTT results, von Willebrand factor panel, Platelet Function Analyzer-100 (PFA-100) results, bone marrow biopsy findings (when available), associated comorbidities or clinical features, family history, and NGS panel results. In select patients, flow cytometric analysis was performed to assess the expression of major platelet glycoproteins. This analysis was conducted on a Navios EX 3L10C flow cytometer (Beckman Coulter, Brea, California, United States) using a custom Platelet Disorders Panel. The panel included the following antibodies: CD42a FITC, CD42b PE, CD41 PE, and CD61 PC5, all from Beckman Coulter. Platelet populations were gated based on forward scatter (FSC) and side scatter (SSC) properties, and their fluorescence intensity was analyzed to determine surface glycoprotein expression. Since LTA was not available at our center, it was not included in the diagnostic workup.

Bleeding severity was categorized as follows: Mucocutaneous bleeding (such as epistaxis, gingival bleeding, purpura, and menorrhagia) was considered mild to moderate, while central nervous system hemorrhage, gastrointestinal bleeding, and bleeding requiring transfusion were classified as severe.

Macrothrombocytopenia was defined as thrombocytopenia with a high MPV (at least one measurement above 10.4 fL, based on the upper limit established in our laboratory) combined with the presence of characteristically large and/or giant platelets on the PBS. Large platelets are significantly larger than typical ones, often about half the size of a normal red blood cell. Giant platelets are as large as or larger than a normal red blood cell. Chronic immune thrombocytopenia (ITP) was defined as thrombocytopenia lasting more than 12 months. Refractory thrombocytopenia was defined as thrombocytopenia that does not respond to at least intravenous immunoglobulin (IVIG) and corticosteroids [5].

### 2.1. Next-Generation Sequencing Analysis

The panel included a total of 14 genes *(RUNX1*, *WAS*, *ADAMTS13*, *ANKRD26*, *CYCS*, *GATA1*, *GP1BA*, *GB1BB*, *GP9*, *ITGA2B*, *ITGB3*, *MASTL*, *MPL*, *MYH9*), covering all coding regions. Except for *MASTL*, all genes are Tier 1 according to the International Society on Thrombosis and Haemostasis (ISTH). *MASTL* is a Tier 2 gene and is reported to be associated with autosomal dominant (AD) thrombocytopenia 2 [6,7]. The IPDs related to the genes and their inheritance patterns are listed in Table 1.

For each patient, 2–5 mL of peripheral blood was collected in tubes containing EDTA to ensure sufficient sample volume for genetic analysis. The automated QIA Symphony DSP DNA Mini Kit (Qiagen, Hilden, Germany) was used to extract genomic DNA from peripheral blood lymphocytes. The extracted DNA samples were either processed immediately or stored at −20 °C for future use. The amplified target genes were sequenced on the MiSeq System (Illumina Inc., San Diego, CA, USA) following the manufacturer’s guidelines. Variants were analyzed using the Qiagen Clinical Insight software 2025 release, which incorporates population frequency databases, global variant databases, and in silico prediction tools. The identified variants were evaluated based on the recommendations from the American College of Medical Genetics. Variants not present in the Leiden Open Variation Database, Human Gene Mutation Database, or ClinVar databases, and not previously reported in the literature, were considered novel. Family studies were not routinely performed in patients with detected variants.

Further genetic testing, such as whole-exome sequencing (WES), was performed on a limited number of patients on a case-by-case basis. This was carried out for those with highly suggestive but unconfirmed clinical phenotypes, especially in cases where features other than thrombocytopenia were prominent.

### 2.2. Statistical Analysis

Mean, standard deviation, median, minimum, maximum values, and percentage were used for descriptive statistics. Analyses were conducted using SPSS (version 26.0).

## 3. Results

### 3.1. Baseline Patient Characteristics

Our study involved 93 unrelated patients, of whom 88 (94.6%) had thrombocytopenia and 5 (5.4%) were suspected of having abnormal platelet function. The median age at presentation, defined as the start of bleeding symptoms and/or the first detection of thrombocytopenia, was 7 years (range, 0–17). Presentation occurred during the neonatal period in 11 patients (11.8%). Overall, 52 patients (55.9%) were male. A history of bleeding was reported in 48 patients (51.6%), with severe bleeding observed in 7 (7.5%). Among the 11 newborns, 4 (36.4%) had a history of bleeding, with 2 (18.2%) experiencing severe bleeding, including intracranial hemorrhage and pulmonary hemorrhage. Consanguinity was present in 24 patients (25.8%), including 15 (16.1%) from first-cousin marriages. A family history of thrombocytopenia was noted in 22 patients (23.7%), one of whom had an uncle with Wiskott–Aldrich syndrome (WAS). Additionally, 22 patients (23.7%) had other associated diseases or features.

Macrothrombocytopenia (35.5%, *n* = 33) and thrombocytopenia with normal platelet size (25.8%, *n* = 24) were the main reasons for NGS panel testing. Other reasons included suspected platelet function disorders (2.2%, *n* = 2), chronic ITP (16.1%, *n* = 15), refractory ITP (15.1%, *n* = 14), confirmation of thrombotic thrombocytopenic purpura (TTP) diagnosis (2.2%, *n* = 2), and Glanzmann thrombasthenia (3.2%, *n* = 3). Table 2 summarizes the detailed laboratory findings, clinical characteristics, and study outcomes based on the reason for panel testing.

### 3.2. Variants Detected by Targeted NGS

A total of 37 variants were identified across 30 patients (32.3% of the cohort). The detected variants, along with details of allelic state, classification, and previously reported cases, are shown in Table 3. Seven of the 11 patients (63.7%) who presented during the neonatal period were found to have variants. In the entire group, each affected patient had one to three variants. At least one variant was detected in all genes on the panel, except for *MPL*, *CYCS*, and *RUNX1*. The most frequently affected gene was *ITGB3* (7 variants, 18.9% of all variants), followed by *GP1BA* (6 variants, 16.2%) and *ADAMTS13* (6 variants, 16.2%). Other notable findings included variants in *ITGA2B* (5 variants, 13.5%) and *ANKRD26* (4 variants, 10.8%). Less common variants were observed in *MYH9* (3 variants, 8.1%), *WAS* (2 variants, 5.4%), and individual variants in *GATA1*, *GP1BB*, *GP9*, and *MASTL* (1 variant each, 2.7%). Fifteen of the 37 variants (40.5%) were novel. Regarding clinical significance, most detected variants were classified as variants of uncertain significance (VUS), accounting for 24 (64.9%). Ten variants (27%) were designated as likely pathogenic (LP), while three variants (8.1%) were confirmed as pathogenic. Genetic analysis showed that most variants were in a heterozygous state (27 variants, 73%). Six variants (16.2%) were identified as being in a homozygous state, including one patient (case No. 7) with two homozygous variants in the *ADAMTS13* gene, and two variants (5.4%) were hemizygous. Additionally, two variants in the *ITGB3* gene were in a compound heterozygous state in case No. 25 (5.4%).

### 3.3. Patients with Macrothrombocytopenia

Among 33 patients with macrothrombocytopenia, 16 genetic variants were identified in 13 cases (39.4%). Variants were present in 10 of the 14 genes included in the panel, and 6 were novel (37.5%) (Table 2). In 9.1% of patients (*n* = 3), co-occurring variants in two genes were detected. Case No. 19 had heterozygous LP variants in both *GP1BB* and *ITGB3*, while case No. 18 had a heterozygous VUS in *GP1BA* and a heterozygous VUS in *ITGA2B*. Neither patient showed any signs of bleeding. A family history of thrombocytopenia was present in both cases. Case No. 10 carried a heterozygous *ANKRD26* VUS and a homozygous *GP9* VUS and showed no signs of bleeding. An isolated heterozygous VUS in ITGB3 was identified in case No. 24, which showed regular CD41/CD61 expression in flow cytometry analysis. Case Nos. 20 and 21, with heterozygous LP and VUS variants in *ITGA2B*, showed large platelets on PBS, with maximum MPV values ranging from 11.7 to 13.2 fL; however, the minimum MPV values for these patients were 6.2–6.5 fL. Flow cytometry analysis of CD41/CD61 expression in case No. 20, who carried the LP *ITGA2B* variant, revealed normal expression levels. Case Nos. 3 and 4, with heterozygous VUS in *ADAMTS13*, showed no evidence of anemia or microangiopathic changes on PBS. ADAMTS13 enzymatic activity was not evaluated in any of the patients.

Considering patients with or without variants, eight patients (25.8%) had additional concomitant diseases. Case No. 28, with a heterozygous VUS in *MASTL*, showed mild bleeding, neurocognitive deficits, and scoliosis, without consanguinity. Interestingly, case No. 13, with a heterozygous LP variant in *GP1BA*, was also diagnosed with glucose-6-phosphate dehydrogenase (G6PD) deficiency. The clinical history included hydrops fetalis, the need for erythrocyte transfusions, and in utero demise of male siblings. Although G6PD levels were very low, the history of transfusions and macrothrombocytopenia prompted preliminary testing for a *GATA1* mutation, which was negative. Subsequent genetic analysis confirmed a hemizygous LP variant in the *G6PD* gene. Case No. 12, a girl who had a heterozygous VUS *GATA1* variant, also had epilepsy.

Among the 20 patients in whom no mutations were identified through initial screening, 5 (25%) exhibited other clinical manifestations: 2 with congenital heart disease (CHD), 1 with short stature, 1 with scoliosis accompanied by a renal anomaly, and 1 with growth hormone deficiency and kyphosis. In this mutation-negative group, seven (35%) reported consanguinity, and seven (35%) had a family history of thrombocytopenia. Finally, one patient was later diagnosed with phytosterolemia following further genetic investigations.

### 3.4. Patients with Normothrombocytopenia

Five variants, including two novel mutations, were identified across four patients (Table 3). Case No. 25 carried a compound heterozygous VUS in *ITGB3*; this patient had a maximum MPV of 7.8 fL and normal-sized platelets on PBS. Case No. 15, with a heterozygous LP *GP1BA* variant, had a maximum MPV of 11 fL, no large or giant thrombocytes on PBS, and normal CD 42a-b expressions. Cases Nos. 1 and 2 had LP and VUS hemizygous *WAS* variants. Case No. 1 experienced neonatal thrombocytopenia and had a maternal uncle with a known WAS history. His MPV ranged from 6.5 to 8.7 fL, and no microthrombocytopenia was seen on PBS. After genetic confirmation of WAS, he underwent hematopoietic stem cell transplantation. Case No. 2, with a VUS hemizygous *WAS* variant, developed thrombocytopenia at nine years old, with MPV values from 6.6 to 9.2 fL, no microthrombocytopenia on PBS, and a history of nephritis.

Among the group, six patients (25%) had comorbidities regardless of variant detection. Notably, case No. 2 had nephritis, and case No. 15 was diagnosed with attention deficit/hyperactivity disorder (ADHD). Focusing on the 20 patients without detected variants, 4 (20%) had additional coexisting conditions: One showed multiple syndromic features, another had CHD, a third experienced sinus vein thrombosis with thyroiditis, and a fourth was diagnosed with an arachnoid cyst. Additionally, consanguinity was noted in five patients (25%), and a positive family history of thrombocytopenia was seen in seven patients (35%). It is also noteworthy that one patient had an uncle with leukemia, suggesting a possible family predisposition.

### 3.5. Patients with Refractory ITP and Chronic ITP

Genetic variants were identified in three genes across both refractory and chronic ITP cohorts. Compared to the chronic ITP group, the refractory ITP group showed a higher percentage of patients with variants (35.7% vs. 20%) and a higher average number of variants per patient (0.36 vs. 0.2). Within the refractory ITP cohort, cases Nos. 8 and 9—who had previously maintained normal platelet counts—were found to carry heterozygous VUS *ANKRD* mutations. Notably, the mother of case No. 8 also carried the same variant but did not have thrombocytopenia. Case No. 9, who experienced an intracranial hemorrhage, was found to have a biallelic pathogenic *ACP5* mutation, indicative of spondyloenchondrodysplasia (SPENCD) upon further genetic testing.

Three patients in the refractory ITP group without detected variants showed other signs of immune dysregulation, such as thyroiditis, positive antinuclear antibodies, and common variable immunodeficiency. However, the variant-positive refractory ITP group did not show additional signs of immune dysregulation.

In the chronic ITP group, case No. 30 carried a heterozygous VUS variant in *MYH9* (MPV: 6.1–12 fL) as well as a heterozygous mutation in *APC*, identified through clinical exome sequencing performed because of hypogammaglobulinemia. This patient had a family history of thrombocytopenia but no personal or family history of colorectal cancer, and responded well to IVIG and eltrombopag. Case No. 27, diagnosed with chronic ITP at age four and who had not needed treatment for 10 years, carries an LP variant in the *ITGB3* gene (MPV: 8.1–9.1 fL).

### 3.6. Patients with Specific Platelet Disorders

Among patients with preliminary diagnoses, all three individuals suspected of having Glanzmann thrombasthenia showed reduced CD41 or CD61 expression on platelets. One of these patients (case No. 11), who had a platelet count as low as 100 × 10^9^/L and an MPV ranging from 8.8 to 11.7 fL, was found to carry heterozygous VUS variants in ANKRD26 and MYH9, along with a homozygous LP variant in ITGB3. This patient also had a history of pulmonary hemorrhage during the neonatal period. Of the remaining patients, one (case No. 26) had a homozygous LP ITGB3 variant, while another had no detectable variants.

Two patients clinically suspected of congenital TTP showed significantly reduced ADAMTS13 enzymatic activity. Consistent with this diagnosis, case No. 6 was found to carry a homozygous pathogenic variant in the ADAMTS13 gene. Interestingly, case No. 7 had two different homozygous VUS variants in the same gene. He experienced an intracranial hemorrhage during the neonatal period. Finally, case No. 5, who had a prolonged PFA-100 closure time without thrombocytopenia and was initially thought to have a platelet function disorder, was found to carry a heterozygous pathogenic variant in the ADAMTS13 gene and showed a mild bleeding phenotype.

## 4. Discussion

Bleeding in IPDs is often moderate but can be severe, affecting the central nervous system or gastrointestinal tract. Accurate diagnosis is crucial for managing bleeding, identifying features and malignancy risks, and pursuing genetic counseling. However, diagnosis is challenging. LTA, the gold standard, is technically demanding, needs large blood volumes, and is hard in children. PFA-100/200 has low sensitivity and specificity, especially with anemia or thrombocytopenia. Flow cytometry helps with some disorders, but it is limited. Many patients remain undiagnosed or misdiagnosed into adulthood [1,4].

NGS represents a shift from first-generation methods like Sanger sequencing, which reads DNA one base at a time but is slow and costly for large projects. NGS, or second-generation sequencing, introduced massively parallel sequencing, drastically reducing time and costs and enabling routine genome analysis. Limitations include short read lengths that challenge analysis of complex regions. Third-generation technologies provide long reads, resolving complex architectures and structural variants more accurately [22]. IPD-focused NGS panels improve diagnostic efficiency by sequencing multiple genes related to a specific phenotype. To standardize genetic testing for IPDs, the ISTH established a three-tier system for ranking clinically significant genes: Tier 1 genes are supported by multiple pedigrees and functional or mouse model data; Tier 2 genes are identified from small pedigrees needing confirmation; functional or animal studies support Tier 3 genes but lack human pedigree validation [23]. Currently, the ISTH lists 62 IPD-associated genes, exceeding those in our panel [6].

Our retrospective study lacked strict genetic testing criteria. The high consanguinity rate (25.8%), family history of thrombocytopenia (23.7%), and overlapping clinical features support our cohort’s relevance for IPD research. Despite a narrower panel scope, we found variants in 32.3% of patients across 11 of 14 genes, excluding *MPL*, *CYCS*, and *RUNX1*, with a high proportion of novel variants (40.5%), and most variants (64.9%) were VUS. Patients with macro- or normothrombocytopenia without identified variants showed notable consanguinity (35% and 25%) and family history (35%), suggesting a genetic basis beyond our current panel. Wang et al. [24] used an 89-gene panel, finding a 35% diagnostic yield for pathogenic variants, often correcting misdiagnoses of ITP. Romasko et al. [25] diagnosed 23.8% of 21 pediatric suspected IPD cases via exome sequencing, finding pathogenic/LP variants and VUS in 52%. They emphasized the need for functional studies, family segregation, and data sharing to reclassify VUS.

Many patients with macrothrombocytopenia in our study exhibit gene variants, including some with two variants, highlighting the genetic diversity of macrothrombocytopenia. Case Nos. 13 and 14 had isolated heterozygous variants in *GP1BA* alongside macrothrombocytopenia, indicating monoallelic Bernard–Soulier syndrome (BSS). Such BSS cases are often diagnosed later and mistaken for ITP. Laboratory tests show decreased or normal responses to ristocetin-induced platelet aggregation and variable GPIb-IX expression. Therefore, genetic testing is recommended to confirm the diagnosis [26].

In the macrothrombocytopenia group, case No. 24 had a heterozygous VUS *ITGB3,* and case Nos. 20 and 21 had heterozygous LP and VUS *ITGA2B* variants. Homozygous mutations in the extracellular domain of the integrin αIIbbeta3 complex cause Glanzmann thrombasthenia, while heterozygous AD mutations in the intracytoplasmic region can cause thrombocytopenia, presenting as macrothrombocytopenia or normothorombocytopenia. Patients show varying glycoprotein expression, with reduced aggregation to ADP and collagen but normal ristocetin agglutination [14,27]. Variants in genes not typically linked to macrothrombocytopenia, like *ADAMTS13* and *MASTL*, were also found. Case Nos. 3 and 4, with heterozygous VUS *ADAMTS13* variants, lacked microangiopathy, suggesting these variants are incidental; further testing is needed. Case No. 28, with a heterozygous *MASTL* VUS, had neurocognitive impairments, scoliosis, and mild bleeding. While MASTL abnormalities are rare, they are increasingly recognized in inherited thrombocytopenia but are not part of a defined syndrome. A severe aplastic anemia case with a heterozygous MASTL mutation has been reported [28]. The complex phenotype in Case No. 28 might be due to another mutation or unrecognized syndromic aspects of *MASTL*, which is still being defined due to its rarity. The diagnosis of phytosterolemia in a patient with macrothrombocytopenia despite no mutations being identified by our panel highlights the importance of PBS evaluation and expanded genetic testing. Phytosterolemia is a rare AR disorder caused by biallelic *ABCG5* or *ABCG8* mutations, leading to phytosterol accumulation. Hematologic features like macrothrombocytopenia, anemia, and stomatocytosis occur in about 30% of pediatric cases [29]. Although not included in our panel, phytosterolemia genes are listed in the ISTH Tier 1 gene list.

While PBS and MPV assessments are used to classify thrombocytopenia, they are often insufficient. For example, our cohort showed that individuals with heterozygous *GP1BA* variants had normal MPV, while those with heterozygous *ITGA2B* variants had large platelets on PBS despite low MPV. Two patients with hemizygous WAS variants did not show microthrombocytopenia on PBS. Factors like analysis timing and anticoagulant type affect MPV reliability, and MPV may be falsely high with microcytic red blood cells [30,31]. Large platelets also appear in secondary thrombocytopenia, and PBS can vary between observers [32]. Therefore, NGS panels offer a more definitive diagnosis for IPDs, detecting mutations even without classical morphology.

Currently, ITP is a diagnosis of exclusion due to a lack of definitive tests. While steroid and IVIG responses may support ITP diagnosis, variable outcomes suggest misdiagnosis or diverse mechanisms. Genetic testing can improve accuracy and guide treatment. Joshi et al. [33] screened 80 adults and children with chronic ITP, identifying pathogenic or LP variants related to IPDs in 10% of patients via WES or a 96-gene panel, and VUS in 15%. When using a 485-gene panel or WES for primary immunodeficiencies, pathogenic or LP variants appeared in 4%, with VUS in 30%. These findings reveal genetic heterogeneity in chronic ITP and suggest that some cases have an underlying genetic cause, needing thorough evaluation. Our findings support genetic testing in chronic and refractory ITP. The higher prevalence of genetic variants in refractory cases (35.7%) vs. chronic (20%) underscores the need for genetic evaluation, especially in refractory patients. Notably, we identified a novel VUS in the *ANKRD26* gene in case No. 8, with refractory ITP, who previously had normal platelet counts. The same variant was found in the patient’s mother, who did not have thrombocytopenia. This shows the difficulty in interpreting VUS, suggesting variable penetrance or involvement of additional genetic or environmental factors, like a “second hit.” These cases emphasize cautious interpretation of genetic results within the broader clinical and family context. Using NGS panels in ITP patients guides treatment, particularly when gene variants linked to myelodysplastic syndromes or cancers are identified. Thrombopoietin receptor agonists (TPO-RAs) are commonly used for refractory ITP but may worsen disease in those with *ANKRD26*, *ETV6*, or *RUNX1* variants. Their use in these cases is limited and should be used cautiously [34]. On the other hand, case No. 30, with a heterozygous VUS *MYH9* variant and a heterozygous *APC* mutation, responded well to IVIG and eltrombopag. In this patient, the *MYH9* variant likely explains thrombocytopenia; the *APC* mutation, possibly incidental, may have broader immunological effects, as suggested by studies on *APC* mutations affecting T-cell function [35]. The patient’s positive response to IVIG and eltrombopag aligns with data on TPO-RA in MYH9-related disorders [34].

## 5. Conclusions

Despite notable progress, diagnosing IPDs genetically remains difficult due to phenotypic overlap, genetic diversity, and the presence of VUS. Combining comprehensive genetic testing with detailed clinical and laboratory phenotyping is crucial for better diagnosis and patient outcomes. Our study shows that incorporating targeted NGS panels into the diagnostic process for suspected IPDs significantly improves clinical practice by providing valuable genetic insights across various phenotypes. NGS helps identify genetic causes and complex or unusual variant combinations. Genetic testing is vital not only for accurate diagnosis but also for risk assessment, including malignancy risks, personalized treatment, genetic counseling, and better patient outcomes. However, limitations of our retrospective study include the panel’s limited gene coverage compared to ISTH guidelines, which may have overlooked relevant variants. The high rate of VUS underscores the ongoing evolution of genetic interpretation. Importantly, NGS results need careful multidisciplinary review to address variant complexity and ethical issues, especially in IPDs with cancer predisposition.

## Figures and Tables

**Table 1:** The covered genes in the targeted NGS panel, associated disorders, and inheritance patterns.

Gene	Associated Disorders	Inheritance
*RUNX1*	Familial platelet disorder with predisposition to acute myeloid leukemia	AD
*WAS*	Wiskott–Aldrich syndrome	XLR
*ADAMTS13*	Thrombotic thrombocytopenic purpura	AR
*ANKRD26*	Autosomal dominant thrombocytopenia 2	AD
*CYCS*	Autosomal dominant thrombocytopenia 4	AD
*GATA1*	X-linked thrombocytopenia with dyserythropoiesis	XLR
*GP1BA*	Bernard–Soulier syndrome	AR
	Mild macrothrombocytopenia	AD
	Platelet-type von Willebrand disease	AD
*GP1BB*	Bernard–Soulier syndrome	AR
	Mild macrothrombocytopenia	AD
*GP9*	Bernard–Soulier syndrome	AR
*ITGA2B*	Glanzmann thrombasthenia	AR
	Platelet-type bleeding disorder 16	AD
*ITGB3*	Glanzmann thrombasthenia	AR
	Platelet-type bleeding disorder 16	AD
*MASTL*	Autosomal dominant thrombocytopenia 2	AD
*MPL*	Congenital amegakaryocytic thrombocytopenia	AR
*MYH9*	MYH9-related disorders	AD

AD: autosomal dominant, AR: autosomal recessive, MYH9: myosin heavy chain 9, NGS: Next-Generation Sequencing, XLR: X-linked recessive. References: [6,7].

**Table 2:** Clinical characteristics, laboratory findings, and outcomes according to the indication for the NGS panel.

	Macrothrombocytopenia	Normothrombocytopenia	Refractory ITP	Chronic ITP	Glanzman	Thrombocyte Function Disorder	Congenital TTP
Patients, %	33, 35.5%	24, 25.8%	14, 15.1%	15, 16.1%	3, 3.2%	2, 2.2%	2, 2.2%
Diagnosis age, mean or median (range)	7 (0–17)	3.5 (0–17)	7.2 ± 4.7 (0.5–17)	7 ± 4.8 (0.5–16)	0.5 ± 0.5 (0–1)	5.8 ± 6 (1.5–10)	0
Bleeding, %	7, 21.4% 2 severe	9, 37.5%	14, 100% 2 severe	12, 80%	3, 100% 2 severe	2, 100%	1, 50% 1 severe
Minimum platelet count (×10^9^/L), mean or median (range)	77 (3–127)	71.2 ± 40.6 (3–110)	4 (2–29)	13 (2–31)	246.7 ± 154.8 (102–410)	307.5 ± 26.2 (289–326)	23.5 ± 19 (10–37)
Minimum MPV, mean or median (range)	9.7 (6.2–16.9)	8.1 ± 1.1 (6.5–10.5)	6.6 (5.3–14.6)	6.6 (4.8–8.1)	7.9 ± 0.8 (7.4–8.8)	7.3 ± 1.4 (6.3–8.3)	5.5 ± 0.1 (5.5–5.6)
Maximum MPV, mean or median (range)	13.2 (10.5–20)	10.3 ± 1.7 (7.4–13.3)	14.4 ± 3 (9.1–20.5)	12.4 ± 2 (9.1–15.8)	10.2 ± 1.3 (9.3–11.7)	8.1 ± 0.3 (7.9–8.3)	12.4 ± 2.5 (10.6–14.1)
Family history of thrombocytopenia, %	12, 36.4%	8, 33.4%	1, 7.1%	1, 6.7%	_	_	_
Consanguinity, %	9, 27.3%	6, 25%	2, 14.3%	2, 13.3%	2, 66.7%	1, 50%	2, 100%
Patients with variants, %	13, 39.4%	4, 16.6%	5, 35.7%	3, 20%	2, 66.7%	1, 50%	2, 100%
Variant per patient, average (range)	0.48 (0–2)	0.21 (0–2)	0.36 (0–1)	0.2 (0–1)	1.3 (0–3)	0.5 (0–1)	1.5 (0–2)

ITP: immune thrombocytopenia, MPV: mean platelet volume, TTP: thrombotic thrombocytopenic purpura.

**Table 3:** Comprehensive overview of identified variants.

Case No.	Gene(s)	Variant (s)	Allelic State	Class.	Clinical Findings	Previously Reported Cases
1	*WAS*	NM_000377.3: c.225del	Hemi	LP	Normothrombocytopenia, maternal uncle with WAS	Novel
2	*WAS*	NM_000377.3: c.280C>T	Hemi	VUS	Normothrombocytopenia, history of nephritis	A case series by Bildik et al. [8] reported an association of this variant with autoimmunity and microthrombocytopenia.
3	*ADAMTS13*	NM_139027.6: c.3794A>T	Het	VUS	Macrothrombocytopenia	Reported in ClinVar; no published case reports are available.
4	*ADAMTS13*	NM_139025.4: c.1310C>G	Het	VUS	Macrothrombocytopenia	Novel
5	*ADAMTS13*	NM_139027.6: c.3178C>T	Het	P	Thrombocyte function disorder	It is one of the most common variants found in a homozygous or compound heterozygous state [9].
6	*ADAMTS13*	NM_139027.6: c.1187G>A	Hom	P	Congenital TTP	Reported in two separate case reports as being linked to a fatal neonatal TTP phenotype [10,11].
7	*ADAMTS13*	NM_139027.6: c.1778C>T	Hom	VUS	Congenital TTP	Novel
	*ADAMTS13*	NM_139027.6: c.496G>A	Hom	VUS		Novel
8	*ANKRD26*	NM_014915.3: c.3451G>T	Het	VUS	Refractory ITP	Novel
9	*ANKRD26*	NM_014915.3: c.93G>A	Het	VUS	Refractory ITP, diagnosed as SPENCD	Reported in ClinVar; no published case reports are available.
10	*ANKRD26*	NM_014915.3: c.4779G>C	Het	VUS	Macrothrombocytopenia	Reported in ClinVar; no published case reports are available.
	*GP9*	NM_000174.5: c.139C>T	Hom	VUS		Described as a new variant in a large international study on Bernard–Soulier syndrome [12].
11	*ANKRD26*	NM_014915.3: c.2527G>A	Het	VUS	Glanzmann thrombasthenia	Novel
	*ITGB3*	NM_000212.3: c.538G>A	Hom	LP		Reported in a patient with Glanzmann thrombasthenia in a compound heterozygous state with another *ITGB3* variant [13].
	*MYH9*	NM_002473.6: c.575C>T	Het	VUS		Reported in ClinVar; no published case reports are available.
12	*GATA1*	NM_002049.4: c.1202C>G	Het	VUS	Macrothrombocytopenia, epilepsy	Novel
13	*GP1BA*	NM_000173.7: c.1795C>T	Het	LP	Macrothrombocytopenia, G6PDH deficiency	Reported in ClinVar; no published case reports are available.
14	*GP1BA*	NM_000173.7: c.1252G>A	Het	VUS	Macrothrombocytopenia	Novel
15	*GP1BA*	NM_000173.7: c.1241_1297del	Het	LP	Normothrombocytopenia	Novel
16	*GP1BA*	NM_000173.7): c.1267A>C	Het	VUS	Refractory ITP	Novel
17	*GP1BA*	NM_000173.7: c.571G>A	Het	VUS	Refractory ITP	Reported in ClinVar; no published case reports are available.
18	*GP1BA*	NM_000173.7: c.176T>C	Het	VUS	Macrothrombocytopenia	Reported in ClinVar; no published case reports are available.
	*ITGA2B*	NM_000419.5: c.2704A>G	Het	VUS		Novel
19	*GP1BB*	NM_000407.5: c.175G>T	Het	LP	Macrothrombocytopenia	Novel
	*ITGB3*	NM_000212.3: c.2278C>T	Het	LP		Reported in a family with autosomal dominant macrothrombocytopenia [14].
20	*ITGA2B*	NM_000419.5: c.3076C>T	Het	LP	Macrothrombocytopenia	Heterozygous variants are observed in families with macrothrombocytopenia and normothrombocytopenia [15,16].
21	*ITGA2B*	NM_000419.5: c.457G>A	Het	VUS	Macrothrombocytopenia	Reported in ClinVar; no published case reports are available.
22	*ITGA2B*	NM_000419.5: c.457G>A	Het	VUS	Chronic ITP	Reported in ClinVar; no published case reports are available.
23	*ITGA2B*	NM_000419.5: p.V359M	Het	LP	Refractory ITP	Novel
24	*ITGB3*	NM_000212.3: c.1576G>C	Het	VUS	Macrothrombocytopenia	Reported in ClinVar; no published case reports are available.
25	*ITGB3*	NM_000212.3: c.985A>G	Comp Het	VUS	Normothrombocytopenia	Reported in patients with Glanzmann thrombasthenia [17].
		NM_000212.3: c.180C>T		VUS		Detected in blood donors as a silent nucleotide variation [18].
26	*ITGB3*	NM_000212.3: c.1594T>C	Hom	LP	Glanzmann thrombasthenia	Reported in a patient with Glanzmann’s thrombasthenia in a compound heterozygous state with another *ITGB3* variant [19].
27	*ITGB3*	NM_000212.3: c.985A>G	Het	LP	Chronic ITP	Reported in patients with Glanzmann thrombasthenia [17].
28	*MASTL*	NM_001172303.3: c.1934C>T	Het	VUS	Macrothrombocytopenia, neurocognitive impairment, scoliosis	Novel
29	*MYH9*	NM_002473.6: c.4270G>A	Het	P	Macrothrombocytopenia	Reported in patients with MYH9-RD and late-onset hearing loss [20,21].
30	*MYH9*	NM_002473.6: c.4624G>A	Het	VUS	Chronic ITP, hypogammaglobulinemia, heterozygous *APC* mutation	Novel

Comp Het: compound heterozygous, G6PDH: glucose-6-phosphate dehydrogenase, Hemi: hemizygous, Het: heterozygous, Hom: homozygous, ITP: immune thrombocytopenia, LP: likely pathogenic, MYH9-RD: MYH9-related disorder, P: pathogenic; SPENCD: spondyloenchondrodysplasia, TTP: thrombotic thrombocytopenic purpura, VUS: variants of uncertain significance.

## Data Availability

The datasets generated and analyzed during the current study are available from the corresponding author on reasonable request.

## Abbreviations

The following abbreviations are used in this manuscript:

aPTT	Activated Partial Thromboplastin Time
AD	Autosomal Dominant
AR	Autosomal Recessive
BSS	Bernard–Soulier Syndrome
CHD	Congenital Heart Disease
G6PDH	Glucose-6-Phosphate Dehydrogenase
IPD	Inherited Platelet Disorder
ISTH	International Society on Thrombosis and Haemostasis
IVIG	Intravenous Immunoglobulin
ITP	Immune Thrombocytopenia
LP	Likely Pathogenic
LTA	Light Transmission Aggregometry
MPV	Mean Platelet Volume
NGS	Next Generation Sequencing
PBS	Peripheral Blood Smear
PFA-100	Platelet Function Analyzer-100
PT	Prothrombin Time
SPENCD	Spondyloenchondrodysplasia
TPO-RA	Thrombopoietin Receptor Agonist
TTP	Thrombotic Thrombocytopenic Purpura
VUS	Variants of Uncertain Significance
WAS	Wiskott–Aldrich Syndrome
WES	Whole-Exome Sequencing
